# Report on the Sanatary Condition of the Labouring Population of Great Britain. Supplementary Report on the Results of a Special Inquiry into the Practice of Interments in Towns

**Published:** 1844-04-01

**Authors:** 


### Report on the Sanitary Condition of the Labouring Population of Great Britain—Supplementary Report on the Results of a Special Inquiry into the Practice of Interment in Towns—made at the request of Her Majesty's Principal Secretary of State for the Home Department. By *Edwin Chadwick,* Esq. Barrister at Law. Presented to both Houses of Parliament by Command of Her Majesty. 8vo. pp. 279.

The General Report on the sanitary condition of the labouring population did not comprise any examination of the evidence as to the effects produced on the public health by the practice of interring the dead amidst the habitations of the town population. We are informed that this omission arose from the subject being considered too great in extent, and too especial in its nature, to allow of satisfactory investigation at the time.

The materials on which the present Report is founded, appear to have been collected from every source where useful information was likely to be obtained—from ministers of religion called upon to perform funereal rites in the poorer districts—from persons of the labouring class—from officers of benefit societies and burial clubs—from undertakers chiefly engaged in the interment of the dead of the labouring classes—from foreigners, as to the various modes of interment in their own countries, and the administrative regulations thereon—and, finally, from eminent physiologists, as to the effects produced on the health of the living by emanations from human remains in a state of decomposition. In this extensive range of inquiry, the moral as well as the physical facts developed, are often exceedingly loathsome, and read a lesson which we trust the Legislature will not be tardy in turning to some good account.

It appears that, although the necessity of removing interments from the midst of towns is very generally admitted on various considerations, yet statements as to the innocuousness of grave-yards, are supported by the general testimony "of a number of medical witnesses of high professional position, by whom it is alleged that the emanations from decomposing human remains do not produce specific disease, and further, that they are not generally injurious." It is justly observed, that the practical consequences of these doctrines extend beyond the present question, and are so important in their effects on the sanitary economy of all towns, as to require and demand careful investigation of the facts on which they are founded.

The first part of the Report is therefore devoted to the examination of the evidence for and against these conclusions, in order to determine the question of the healthiness or unhealthiness of inter-mural interments, in which an effort is made to trace the distinct effects produced by emanations from bodies in a state of decay and putrefaction.

The inquiry is subsequently carried forward to determine the injuries to the health of the survivors occasioned by the delay of interments, in which the moral evils produced by the practice are powerfully brought out.

The remainder of the Report, although highly interesting, is less within our province, as it relates chiefly to the expenses of burial proving a pregnant cause of delay—to the remedies proposed to obviate the evil, with lengthened details as to the practicability of ensuring for the public superior interments at greatly reduced expenses: enforced by examples of what has been effected in Germany, France, and America, by successful legislation for the improvement of the practice of interment—and, lastly, to the result of experience in respect to sites of places of burial, the sanitary precautions necessary in respect to them, and the extent of burial-grounds at present existing in the Metropolis.

The concluding sections are devoted to a consideration of the moral influences of seclusion from thronged places and of decorative improvements in national cemeteries, and the necessity and nature of the superior agency requisite for private and public protection in respect to interments; in which is included, a scheme for the appointment of a staff of ten inspectors of public health, varying in rank and emolument from that of inspector-general of hospitals in the army and fleet, to that of regimental surgeons.

In the Appendix will also be found much valuable evidence on the regulations in force at Munich and Frankfort; on the existing defective arrangements for the verification of causes of death; on cases of infanticide committed partly for the sake of burial money; and a view of the extent of intra-mural burial-ground provided, as compared with the extent of extra-mural burial-ground required for the metropolis, &c.

With this short summary of the general scope and bearing of the Report we may now address ourselves more especially to the evidence on the effects produced by emanations from bodies in a state of decay, and the consequent evils to be traced to delayed burial and interment in the midst of the habitations of the population.

The writer has not apparently gone " far a field " for evidence in favour of the innocuousness of putrefaction, consequently the testimony adduced of Dr. Warner of Boston, M. Parent Duchâtelet and Dr. Dunglison, not very conclusive in its nature, is very easily set aside : for instance, although the latter gentleman maintains that " we have no satisfactory proof that malaria ever arises from animal putrefaction singly," yet, unwilling to trust even this negative position alone, it is quickly guarded by the following admission.

" In stating the opinion that putrefaction singly does not occasion malarious disease, we do not mean to affirm that air highly charged with putrid miasmata may not, in some cases, powerfully impress the nervous system so as to induce syncope and high nervous disorder; or that, when such miasmata are absorbed by the lungs in a concentrated state, they may not excite putrid disorders, or dispose the frame to unhealthy erysipelatous affections. On the contrary, experiment seems to have shown that they are deleterious when injected; and cases are detailed in which, when exhaled from the dead body, they have excited serious mischief in those exposed to their action." 3.

So in reference to Parent Duchâtelet's statements, where he cites instances of the exhumation of bodies in an advanced state of decomposition, without any injurious consequences being experienced by the persons engaged in conducting them—the accuracy of M. Duchâtelet's evidence is very quickly disposed of in a Report of Dr. V. A. Riecke, of Stuttgart, on the Influence of Putrefactive Emanations on the Health of Man, in which the medical evidence of this class is closely investigated.—Dr. Riecke observes—

" When Parent Duchâtelet appeals to and gives such prominence to the instance of the disinterments from the churchyard of St. Innocens, and states that they took place without any injurious consequences, although at last all precautions in the mode of disinterring were thrown aside, and that it occurred during the hottest season of the year, and therefore that the putrid emanations might be believed to be in their most powerful and injurious state, I would reply to this by asking the simple question, what occasion was there for the disinterment? Parent Duchâtelet maintains complete silence on this point; but to me the following notices appear worthy of attention. In the year 1554, Houlier and Fernel, and in the year 1738, Lemery, Geoffroy, and Hunaud, raised many complaints of this churchyard; and the two first had asserted that, during the plague, the disease had lingered longest in the neighbourhood of the Cimetière de la Trinité, and that there the greatest number had fallen a sacrifice. In the years 1737 and 1746, the inhabitants of the houses round the churchyard of St. Innocens complained loudly of the revolting stench to which they were exposed. In the year 1755 the matter again came into notice: the inspector who was intrusted with the inquiry, himself saw the vapour rising from a large common grave, and convinced himself of the injurious effects of this vapour on the inhabitants of the neighbouring house. ' Often,' says the author of a paper which we have before often alluded to, ' the complexions of the young people who remain in this neighbourhood grow pale. Meat sooner becomes putrid there than elsewhere, and many persons cannot get accustomed to these houses.' In the year 1779, in a cemetery which yearly received from 2000 to 3000 corpses, they dug an immense common grave near to that part of the cemetery which touches upon the Rue de la Lingerie. The grave was 50 feet deep, and made to receive from 1500 to 1600 bodies. But in February, 1780, the whole of the cellars in the street were no longer fit to use. Candles were extinguished by the air in these cellars; and those who only approached the apertures were immediately seized with the most alarming attacks. The evil was only diminished on the bodies being covered with half a foot of lime, and all further interments forbidden. But even that must have been found insufficient, as, after some years, the great work of disinterring the bodies from this churchyard was determined upon. This undertaking, according to Thouret's report, was carried on from December, 1785, to May, 1786; from December, 1786, to February, 1787; and in August and October of the same year: and it is not unimportant to quote this passage, as it clearly shows how little correct Parent Duchâtelet was in his general statement, that those disinterments took place in the hottest seasons of the year. It is very clear that it was exactly the coldest seasons of the year which were chosen for the work; and though in the year 1787 there occurs the exception of the work having been again begun in August, I think it may be assumed that the weather of this month was unusually cold, and it was therefore thought the work might be carried on without injurious effects. It does not, however, appear to have been considered safe to continue the work at that season, since the report goes on to state that the operations were again discontinued in September.

" Against those statements of Parent Duchâtelet, as to the innocuousness of the frequent disinterments in Père la Chaise, statements which are supported by the testimony of Orfila and Ollivier, in regard to their experience of disinterments, I would here place positive facts, which are not to be rejected. ' I,' also remarks Duvergie, ' have undertaken judicial disinterments, and must declare that, during one of these disinterments at which M. Piedagnel was present with me, we were attacked with an illness, although it was conducted under the shade of a tent, through which there was passing a strong current of wind, and although we used chloride of lime in abundance. M. Piedagnel was confined to his room for six weeks.' Apparently, Duvergie is not far wrong when he states his opinion that Orfila had allowed himself to be misled by his praiseworthy zeal for the more general recognition of the use of disinterments for judicial purposes, to understate the dangers attending them, as doubtless he had used all the precautions during the disinterments which such researches demand; and to these precautions (which Orfila himself recommended) may be attributed the few injurious effects of these disinterments. It, however, deserves mentioning, that, if Orfila did undertake disinterments during the heat of summer, it must have been only very rarely; at least, amongst the numerous special cases which he gives, we find only two which took place in July or August, most of the cases occurred in the coldest season of the year. I cannot refrain from giving, also, the information which Fourcroy gained from the grave-diggers of the churchyard of St. Innocens. Generally they did not seem to rate the danger of displacing the corpses very high: they remarked, however, that some days after the disinterment of the corpses the abdomen would swell, owing to the great development of gas; and that if an opening forced itself at the navel, or anywhere in the region of the belly, there issued forth the most horribly smelling liquid and a mephitic gas; and of the latter they had the greatest fear, as it produced sudden insensibility and faintings. Fourcroy wished much to make further researches into the nature of this gas, but he could not find any grave-digger who could be induced by an offered reward to assist him by finding a body which was in a fit state to produce the gas. They stated, that, at a certain distance, this gas only produced a slight giddiness, a feeling of nausea, languor, and debility. These attacks lasted several hours, and were followed by loss of appetite, weakness and trembling. ' Is it not very probable,' says Fourcroy, ' that a poison so terrible that when in a concentrated state, it produced sudden death, should, even when diluted and diffused through the atmosphere, still possess a power sufficient to produce depression of the nervous energy and an entire disorder of their functions? Let any one witness the terror of these grave-diggers, and also see the cadaverous appearance of the greatest number, and all the other signs of the influence of a slow poison, and they will no longer doubt of the dangerous effects of the air from churchyards on the inmates of neighbouring houses.'" 6.

"An eminent surgeon" expresses his belief that no injury resulted from emanation from decomposing remains—*but* mentioning an instance in proof, where he suffered no injury, he admitted that his assistant was immediately after taken ill, and long remained so!

To prove the innocuousness of emanation from human remains on the general health, evidence of another class is adduced, consisting of persons acting as keeepers of dissecting-rooms, and grave-diggers, and the undertakers' men, "who, it is stated, have pursued their occupations for long periods, and have nevertheless maintained robust health."

Investigation brings out some curious facts indicative of the fallacy of this class of evidence. "Professor Owen mentions a report of a robust man, the keeper of a dissecting-room, who appeared to be in florid health (which, however, proved to be less sound than he flattered himself) who was unconscious of having sustained any injury from the occupation—but it turned out, on inquiry, that he had always had the most offensive and dangerous work done by an inferior assistant!

If mere fattening were an evidence of occupation agreeing with a man, many of our readers might recall to mind the huge and portly figure of "George," of Windmill Street memory, who, some twenty years ago, seemed to bid defiance to all external agents—but, peace to his ashes!—the veriest scarecrows of leanness would have been loath to exchange their skins for his gloated rotundity!

So, frequently the sextons of grave-yards, who are robust men, attest the salubrity of the place, but on examining the grave-diggers, it appears that, as a class, they are unhealthy and cadaverous, and, notwithstanding precautions, often suffer severely on re-opening graves, and that their lives are frequently cut short by the work. There are florid and robust undertakers, but, as a class, with all the precautions they use, they are unhealthy, and one of them, of considerable business states, "in nine cases out of ten, the undertaker who has much to do with the corpse is a person of cadaverous hue, and you *may almost always tell him wherever you see him*!"

Again—

"Medical papers have been written in this country and on the continent to show that the exposure of workmen to putrid emanations in the employment of sewer cleansing has no effect on the general health; and when the employers of the labourers engaged in such occupations are questioned on the subject, their general reply is, that their 'men have nothing the matter with them:' yet when the *class* of men who have been engaged in the work during any length of time are assembled; when they are compared with classes of men of the same age and country, and of the like periods of service in other employments free from such emanations, or still more when they are compared with men of the same age, coming from the purer atmosphere of a rural district, the fallacy is visible in the class, in their more pallid and shrunken aspect—the evidence of languid circulation and reduced 'tone,' and even of vitality—and there is then little doubt of the approximation given me by an engineer who has observed different classes of workmen being correct, that employment under such a mephitic influence as that in question ordinarily entails a loss of at least one-third of the natural duration of life and working ability." 9.

Abundant evidence is, in fine, adduced to leave no shadow of doubt on any rational mind, that all emanations from the decay and putrefaction of animal bodies are injurious in the highest degree—deteriorating health, and greatly shortening the average duration of life. These influences are not the less fatal that the effects do not invariably, or even generally, appear in the form of acute disease—indeed, were it so, large masses of the population who have lived under their influence must have been exterminated. It is correctly remarked, that the injurious effects of diluted emanations are constantly traceable, not in constitutional disturbance at any one time—their effect, on the strong, is perceptible only over a space of time, and in a general depression of health, and a shortened period of existence.

The fact of the general offensiveness of such emanations, is adduced by Dr. Riecke also as an evidence of their injurious quality.

"Another circumstance which must awaken in us distrust of putrid emanations, is the powerful impression they make on the sense of smell. It certainly cannot be far from the truth to call the organ of smell the truest sentinel of the human frame. 'Many animals,' observes Rudolphi, 'are entirely dependent on their sense of smell for finding out food that is not injurious; where their smell is injured, they are easily deceived, and have often fallen a sacrifice to the consequent mistakes.' Amongst all known smells, there is, perhaps, no one which is so universally, and to such a degree revolting to man, as the smell of animal decomposition. The roughest savage, as well as the most civilised European, fly with equal disgust from a place where the air is infected by it. If an instinct ever can be traced in man, certainly it is in the present case: and is instinct a superfluous monitor exactly in this one case? Can instinct mislead just in this one circumstance? Can it ever be, that the air which fills us with the greatest disgust, is the finest elixir of life, as Dumoulins had the boldness to maintain in one of his official reports? Hippolyte Cloquet, in his Osphrestologie has attempted to throw some light on the effect of smell on the human frame, and though we must entirely disregard many of the anecdotes which he has blended into his inquiry, yet the result remains firmly proved that odours in general exert a very powerful influence on the health of men, and that all very acutely impressing smells are highly to be suspected of possessing injurious properties." 15.

Mr. Chadwick's general conclusions from the foregoing part of the Report is as follows :—

"The injurious effect of the exhalations from the decomposition in question, upon the health and life of man is proved by a sufficient number of trustworthy facts;

"That this injurious influence is by no means constant, and depends on varying and not yet sufficiently explained circumstances;

"That this injurious influence is manifest in proportion to the degree of concentration of putrid emanations, especially in confined spaces; and in such cases of concentration the injurious influence is manifest in the production of asphyxia and the sudden and entire extinction of life;

"That, in a state less concentrated, putrid emanations produce various effects on the nerves of less importance, as fainting, nausea, head-ache, languor;

"These emanations, however, if their effect is often repeated, or if the emanations be long applied, produce nervous and putrid fevers; or impart to fevers, which have arisen from other causes, a typhoid or putrid character;

"Apparently they furnish the principal cause of the most developed form of typhus, that is to say, the plague (*Der Bubonenpest.*) Besides the products of decomposition, the contagious material may also be active in the emanations arising from dead bodies." 23.

How far the last paragraph may be borne out is a question upon which we will not now enter—but we are well satisfied of the general truth of these conclusions; and, although from the nature of the emanations, ever in operation with many other causes and influences, the attainment of any definite or proximate evidence of the extent of the operation of those emanations on the health of the population must be nearly hopeless in crowded districts, enough has been obtained to shew that they are among the most fatal of the deleterious influences which waste and depress the vital powers, and fearfully abridge the natural term or duration of life. We shall presently find that the mere exposure during a few days in the close and crowded tenements of the poor to the emanations of a corpse, leads to the most fatal consequences; but, in the meantime, let us examine how far a superstratum of earth, such as is laid over the bodies in our metropolitan churchyards, shields the surrounding population from the effects of putrefaction after burial.

We find that in the Metropolis, on "spaces of ground which do not exceed 203 acres, closely surrounded by the abodes of the living, layer upon layer, each consisting of a population numerically equivalent to a large army of 20,000 adults and nearly 30,000 youths and children, is every year imperfectly interred. Within the period of the existence of the present generation, upwards of a *million of dead must have been interred in the same spaces.*" Again, "a layer of bodies is stated to be about seven years in decaying in the Metropolis: to the extent that this is so, the decay must be by the conversion of the remains into a gas, and its escape as a miasma, of many times the bulk of the body that has disappeared."

This septennial process of clearing the ground by the conversion of solids into gases gives such a vast generation of the latter, which must be continually finding its way into the air we breathe, and the water we drink, that, unless they be wholly innocuous, alarm may well be felt at this increasing evil. But we cannot blind ourselves by any fallacy, to the deleterious nature of these emanations, for, although in a vast metropolis like this, where leakage from cesspools and leakage from coal-gas pipes alike exist, and where in fact there is every possible complication of mal-odorous emanations, popular perceptions as well as chemical analysis may be baffled by the combination of all, in affixing to any one its true amount of mischief, and the Commissioners of Sewers, the gas manufacturers, undertakers, &c. may each contend it is their neighbour, yet facts are sufficiently abundant to leave no doubt as to the effects upon the life and health of the surrounding population from the present system of burial.

At Ciudad Rodrigo—to take this part of the subject in its simplest form—where Sir James Macgrigor states 20,000 dead bodies were put into the ground within the space of a few months; all those exposed to the emanations from the soil, and obliged to drink the water from the sunk wells *were affected by malignant and low fevers and dysentery,* or fevers frequently putting on a dysenteric character. And this, be it remembered, was in an open country.

In reference to the wells, it is obvious how widely this source of evil must extend to all classes. In vain are new squares and splendid mansions built for the wealthy and the noble, farther and farther apart from the densely-populated quarters, where narrow streets, close air, and squalid poverty combine to generate sickness, and mingle poison with the atmosphere—even in those places, where the air is less tainted, they can have no security that the water which they drink does not carry with it the seeds of disease and death, polluted with a churchyards' foul corruption and loathsome gases. We do not believe that this argument is required to induce our Legislators to give prompt attention to this health-destroying evil, and devise permanent and efficient means for its removal. Nevertheless, it is right that it should be known that all—from the highest to the lowest—have a vital interest in the remedy which has been too long delayed for this deplorable state of things.

In consequence of various investigations in France, a law was passed prohibiting the opening of wells within 100 metres of any place of burial, but this distance is now found to be insufficient for deep wells, which, on examination, have been proved to be polluted at a distanee of from 150 to 200 metres. In some parts of Germany there are laws equally prohibiting the opening of wells nearer than 300 feet.

It has been alleged that were bodies buried deeper all the present evils to the surrounding population might be obviated. But, however deep a body may be buried it will decay, and so certain as a body has wasted, and by so much disappeared, so certain is the fact that a deleterious gas has escaped. Dr. Reid detected the escape of deleterious miasma from graves of more than 20 feet deep. Another consideration presents itself—if the interments be so deep as to impede escapes at the surface, there is only the greater danger of escape by deep drainage and the pollution of springs.

That this pollution of the springs is no imaginary evil or remote danger we have sufficient evidence. Professor Brande states, that he has "frequently found the well-water of London contaminated by organic matters and ammoniacal salts,"—and he refers to one instance, "where the water of a well near a churchyard had acquired both odour and colour from the soil." Although some of the best springs in the Metropolis are said to be, fortunately, of a depth not likely to be considerably affected by the filtration of these gases through the layers of earth—yet how much that is deleterious may find its way, from these sources, even into the best springs, which escapes the chemist's tests and all his powers of analysis.

It would appear that the Houses of Parliament enjoy no enviable site from this cause. Dr. Reid reports—

"Where the drainage of the district in which the church may be placed is of an inferior description, and liable to be impeded periodically by the state of the tide, as in the vicinity of the Houses of Parliament, where all the drains are closed at high water, the atmosphere is frequently of the most inferior quality. I am fully satisfied, for instance, not only from my own observation, but from different statements that have reached me, and also from the observations of parties who have repeatedly examined the subject at my request, that the state of the burying-ground around St. Margaret's church is prejudicial to the air supplied at the Houses of Parliament, and also to the whole neighbourhood. One of them, indeed, stated to me lately that he had avoided the churchyard for the last six months, in consequence of the effects he experienced the last time he visited it. These offensive emanations have been noticed at all hours of the night and morning; and even during the day the smell of the churchyard has been considered to have reached the vaults in the House of Commons, and traced to sewers in its immediate vicinity. When the barometer is low, the surface of the ground slightly moist, the tide full, and the temperature considerable—all which circumstances tend to favour the evolution of effluvia both from the grave-pits and the drains—the most injurious influence upon the air is observed. In some places not far from this churchyard fresh meat is frequently tainted in a single night, on the ground-floor, in situations where at a higher level it may be kept without injury for a much longer period. In some cases, in private houses as well as at the Houses of Parliament, I have had to make use of ventilating shafts or of preparations of chlorine, to neutralize the offensive and deleterious effects which the exhalations produced, while, on other occasions, their injurious influence has been abundantly manifested by the change induced in individuals subjected to their influence on removing to another atmosphere. No grievance, perhaps, entails greater physical evils upon any district than the conjoined influence of bad drainage and crowded churchyards; and until the drainage of air from drains shall be secured by the process adverted to in another part of this work, or some equivalent measures, they cannot be regarded as free from a very important defect." 29.

Nor should it be forgotten that the effects of the escape of these deleterious gases is not the less when scattered over a town, because in a multitude of scents they escape observation.

We deem it unnecessary to quote any of the very convincing examples of persons and whole families whose health, and, in some instances, whose lives, are shown to have been undoubtedly destroyed by a residence in the neighbourhood of the churchyards of the metropolis. These, indeed, were scarcely needed to fully bear out the following conclusions.

" That inasmuch as there appear to be no cases in which the emanations from human remains in an advanced stage of decomposition are not of a deleterious nature, so there is no case in which the liability to danger should be incurred either by interment (or by entombment in vaults, which is the most dangerous) amidst the dwellings of the living, it being established as a general conclusion in respect to the physical circumstances of interment, from which no adequate grounds of exception have been established;—

" That all interments in towns, where bodies decompose, contribute to the mass of atmospheric impurity which is injurious to the public health." 31.

The inquiry into the injuries, physical and moral, to the survivors occasioned by the *delay of interments*, discloses a picture of misery and sickness at once hopeless and helpless, and of moral prostration and recklessness, such as no period of barbarism probably ever surpassed, and furnishes a bitter moral to our boasted civilization.

This part of the Report begins by premising that—

" In a large proportion of cases in the metropolis, and in some of the manufacturing districts, one room serves for one family of the labouring classes: it is their bed room, their kitchen, their wash-house, their sitting room, their dining room; and, when they do not follow any out-door occupation, it is frequently their work room and their shop. In this one room they are born, and live, and sleep, and die amidst the other inmates." 31.

And the statistical inquiry made at Lord Sandon's expence by Mr. Wild, the Secretary of the Statistical Society, as to the condition of the working classes, shows that, in the parish of St. George's, Hanover Square, and in the immediate vicinity therefore of the most opulent residences of the metropolis, 1465 families had for their residence 2175 rooms, and of these 929 *families* had but a single room, and 623 only one bed! Need we wonder, then, at the statement which follows as a corollary, that out of 5945 persons 839 were found to be ill, and yet the season was not unhealthy. One family in eleven had a third room (but not unoccupied) in which to place a corpse. This however turns out to be a favourable view —from an examination made by a Committee of the Statistical Society into the condition of the poorer classes in the borough of Marylebone, it appears that not one family in a hundred had a third room. Moreover, it was found that 159 families and 196 single persons occupied *part of a room*, and 382 families and 56 single persons occupied one room each.

In this state of things let us see what takes place when sickness ends in death. In answer to the question of how long the dead body is retained in the room beside the living? Mr. Leonard, the medical officer of the parish of St. Martin's in the Fields, replies—

"If the person has subscribed to a club, or the friends are in circumstances to afford the expense of the funeral, it takes place, generally, on the following Sunday, if the death has occurred early in the week; but if towards the end of the week, then it is sometimes postponed till the Sunday weeek after, if the weather permit; in one case it was twelve days. In the other cases I have known much opposition to removal till after a subscription had been collected from the affluent neighbours; and in some instances, after keeping the body several days, I have been applied to to present the case to the relieving officer, that it might be buried by the parish." 32.

"In what condition is the corpse usually, or frequently, retained?—Amongst the Irish, it does not signify of what disease the person may have died, it is retained often for many days, laid out upon the only bed, perhaps, and adorned with the best they can bestow upon it, until the *coronach* has been performed. Thus fevers and other contagious diseases are fearfully propagated. I remember a case of a body being brought from the Fever Hospital to Bullin-court, and the consequences were dreadful; and this spring I removed a girl, named Wilson, to the infirmary of the workhouse, from a room in the same court. I could not remain two minutes in it; the horrible stench arose from a corpse which had died of phthisis twelve days before, and the coffin stood across the foot of the bed, within eighteen inches of it. This was in a small room not above ten feet by twelve feet square, and a fire always in it, being (as in most cases of a like kind) the only one for sleeping, living, and cooking in. I mention these as being particular cases, from which most marked consequences followed; but I have very many others, in which the retention of the body has been fraught with serious results to the survivors.

"Will you describe the consequences of such retention?—Upon the 9th of March, 1840, M——— was taken to the Fever Hospital. He died there, and without my knowledge the body was brought back to his own room. The usual practice, in such cases, is to receive them into a lock-up room, set apart for that purpose in the workhouse. I find that upon the 12th his step-son was taken ill. He was removed immediately to the Fever Hospital. Upon the 18th the barber who shaved the corpse was taken ill, and died in the Fever Hospital, and upon the 27th another step-son was taken ill, and removed also.

"Upon the 18th of December, 1840, I——— and her infant were brought, ill with fever, to her father's room in Eagle-court, which was ten feet square, with a small window of four panes; the infant soon died. Upon the 15th of January, 1841, the grandmother was taken ill; upon the 2nd of February the grandfather also. There was but one bedstead in the room. They resisted every offer to remove them, and I had no power to compel removal. The corpse of the grandmother lay beside her husband upon the same bed, and it was only when he became delirious and incapable of resistance that I ordered the removal of the body to the dead-room, and him to the Fever Hospital. He died there, but the evil did not stop here: two children, who followed their father's body to the grave, were, the one within a week and the other within ten days, also victims to the same disease. In short, five out of six died." 33.

"Comparing the effects of the practice of retaining the bodies before interment, with the effects of emanations from the dead after interment, when buried in crowded districts, which appears to you to be the most pernicious practice?—When a body is retained in a small room, badly ventilated, and often with a fire in it, the noxious gases evolved in the process of decomposition are presented to persons exposed to them in a highly concentrated form, and if their health is in a certain state favourable to receive the contagion, the effect is immediate." 34.

Again, the medical officer of the Whitechapel Union, who is called upon to attend the dock-labourers, navigators, and bricklayers, chiefly, on being asked what was the general condition of a family among these classes when death occurred, states—

"Nearly the whole of the labouring population there have only one room. The corpse is therefore kept in that room where the inmates sleep and have their meals. Sometimes the corpse is stretched on the bed, and the bed and bed-clothes are taken off, and the wife and family lie on the floor. Sometimes a board is got on which the corpse is stretched, and that is sustained on tressels or on chairs. Sometimes it is stretched out on chairs. When children die, they are frequently laid out on the table."

"What is the usual length of time that the corpse is so kept?—The time varies according to the day of the death. Sunday is the day usually chosen for the day of burial. But if a man die on the Wednesday, the burial will not take place till the Sunday week following. Bodies are almost always kept for a full week, frequently longer." 35.

And, turning from the physical evils in all their proteian forms, engendered and nourished until they bring forth their fruits in pestilence and death, if we inquire into the moral effects of this revolting and compulsory jostling together of the dead and the living—where death is robbed of all its solemnity and purifying influences by the imperious necessity of bringing it in daily and hourly contact with the cares and the wants of mere animal life—we find the unnatural union produces a hideous familiarity, and a callousness of feeling, which prepares men for the lowest depths of crime and degradation. Never are Nature's laws, thus violently outraged without retribution—death was no doubt intended to inspire us with solemn thoughts, not unmingled with awe, and to exercise upon our hearts a chastening influence—an influence at once tranquillizing and elevating. "When thou hast wept awhile," says Jeremy Taylor, "compose the body to burial; which, that it be done gravely, decently, and charitably, we have the examples of all nations to engage us, and of all ages of the world to warrant—so that it is against common honesty and public fame and reputation not to do this office."

With this feeling at our hearts, let us turn once more to the present state of the labouring population of this metropolis, and see how far respect to the dead can be shown by these more than wretched inhabitants. Mr. Bestow, the relieving officer of Bethnal-Green, who is called upon to visit the abodes of this class, who on the occurrence of death fall into a state of destitution, which speaks of a lower depth still than their ordinary misery.

"Is the corpse generally kept in the living or in the working room?—In the majority of cases the weavers live and work in the same room; the children generally sleep on a bed pushed under the loom. Before a coffin is obtained, the corpse is generally stretched on the bed where the adults have slept. It is a very serious evil in our district, the length of time during which bodies have been kept under such circumstances. I have frequently had to make complaint of it. We are very often complained to by neighbours of the length of time during which the bodies are kept. We have very often had disease occasioned by it. I have known, in one case, as many as eight deaths, from typhus fever, follow one death; there were five children and two or three visitors whose illness and deaths were ascribed to the circumstance." 36.

A step further, and we read the moral effects.

"Do you observe any peculiarity of habit amongst the lower classes accompanying this familiarity with the remains of the dead?—What I observe when I first visit the room is a degree of indifference to the presence of the corpse: the family is found eating or drinking or pursuing their usual callings, and the children playing. Amongst the middle classes, where there is an opportunity of putting the corpse by itself, there are greater marks of respect and decency." 35.

Another witness states—

"When deaths occur in this class the corpse cannot be laid out without occupying the space where the family have to work (the father or mother weaving, and children winding or rendering other assistance), or in the room where they live and eat. This, I am of opinion, has a very debasing effect on the morals of this class of the community, making especially the rising generation so familiar with death that their feelings are not hurt by it: it has also a very injurious physical effect, frequently propagating disease in a rapid manner and to an immense extent." 41.

The truth of the following observations does not admit of doubt.

"The mental effects on the elder children or members of the family of the retention of the body in the living room, day after day, and during meal times, until familiarity is induced,—retained, as the body commonly is, during all this time in the *sordes* of disease, the progress of change and decomposition disfiguring the remains and adding disgust to familiarity,—are attested to be of the most demoralizing character. Such deaths occur sooner or later in various forms in every poor family; and in neighbourhoods where there are no sanitary regulations, where they are ravaged by epidemics, such scenes are doubly familiar to the whole population." 45.

Great as may be the physical evils to the survivors, and they are so great as scarcely to admit of exaggeration; the mental pain and the moral evil attendant on the practice of the long retention of the body in the rooms in use, and amidst the living, is yet more deplorable; and we could have desired that these occupied a somewhat more prominent place in the present Report. They are by no means overlooked however, although in the evidence they are generally noticed only incidentally. It is remarked very justly that—

"The duty which attaches to male relations, or which a benevolent pastor, if there were the accommodation, would exercise on the occurrence of the calamity of death to any member of a family, is to remove the sensitive and the weakly from the spectacle, which is a perpetual stimulus to excessive grief, and commonly a source of painful associations and visible images of the changes wrought in death, to haunt the imagination in after-life. When the dissolution has taken place under circumstances such as those described, it is not a few minutes' look after the last duties are performed and the body is composed in death and left in repose, that is given to this class of survivors, but the spectacle is protracted hour after hour through the day and night, and day after day, and night after night, thus aggravating the mental pains under varied circumstances, and increasing the dangers of permanent bodily injury. The sufferings of the survivors, especially of the widow of the labouring classes, are often protracted to a fatal extent. To the very young children, the greatest danger is of infection in cases of deaths from contagious and infectious disease. To the elder children and members of the family and inmates, the moral evil created by the retention of the body in their presence beyond the short term during which sorrow and depression of spirits may be said to be natural to them is, that familiarity soon succeeds, and respect disappears." 44.

"Astonishment is frequently excited by the cases which abound in our penal records indicative of the prevalence of habits of savage brutality and carelessness of life amongst the labouring population; but crimes, like sores, will commonly be found to be the result of wider influences than are externally manifest; and the reasons for such astonishment, will be diminished in proportion as those circumstances are examined, which influence the minds and habits of the population more powerfully than precepts or book education. Among these demoralizing circumstances, which appear to be preventible or removable, are those which the present inquiry brings to light. Disrespect for the human form under suffering, indifference or carelessness at death,—or at that destruction which follows as an effect of suffering—is rarely found amongst the uneducated, unconnected with a callousness to others' pain, and a recklessness about life itself. A known effect on uneducated survivors of the frequency of death amongst youth or persons in the vigour of life, is to create a reckless avidity for immediate enjoyment. Some examples of the demoralization attendant on such circumstances cannot but be apparent in the evidence arising in the course of this inquiry into other practices connected with interments.

"On submitting the above to a friend, a clergyman, whose benevolence has carried him to alleviate the sufferings in several hundred death-bed scenes in the abodes of the labouring classes, and who has been present, perhaps, at every death in his own flock, in a wretchedly crowded parish, he writes in the following terms his confirmation:—

"'The whole of this I can testify, from personal knowledge, to be just. With the upper classes, a corpse excites feelings of awe and respect; with the lower orders, in these districts, it is often treated with as little ceremony as the carcase in a butcher's shop. Nothing can exceed their desire for an imposing funeral; nothing can surpass their efforts to obtain it; but the deceased's remains share none of the reverence which this anxiety for their becoming burial would seem to indicate. The inconsistency is entirely, or at least in great part, to be attributed to a single circumstance—that the body is never absent from their sight—eating, drinking, or sleeping, it is still by their side; mixed up with all the ordinary functions of daily life, till it becomes as familiar to them as when it lived and moved in the family circle. From familiarity it is a short step to desecration. The body, stretched out upon two chairs, is pulled about by the children, made to serve as a resting-place for any article that is in the way, and is not seldom the hiding-place for the beer-bottle or the gin if any visitor arrives inopportunely. Viewed as an outrage upon human feeling, this is bad enough; but who does not see that when the respect for the dead, that is, for the human form in its most awful stage, is gone, the whole mass of social sympathies must be weakened—perhaps blighted and destroyed? At any rate it removes that wholesome fear of death which is the last hold upon a hardened conscience. They have gazed upon it so perpetually, they have grown so intimate with its terrors, that they no longer dread it, even when it attacks themselves, and the heart which vice has deadened to every appeal of religion is at last rendered callous to the natural instinct of fear.'

"That it is possible by legislative means to stay the progress of this dreadful demoralization, which must, if no further heed be taken of it, go on with the increased crowding of an increasing population; that it is possible to abate the mental and physical suffering; to extend to the depressed urban districts an acceptable and benign and elevating influence on such impressive occasions; may be confidently affirmed, and will in a subsequent stage of this Report be endeavoured to be shown by reference to actual examples of successful measures." 46.

With these well-timed remarks we will leave this part of the Report. It is unnecessary to dwell upon a fact so generally known—that in all countries, in proportion to the want of respect for the remains of the dead, is the disregard for human life, and that any causes leading to the universal desecration of those remains throughout the labouring classes, must in the same degree tend to render life insecure, to loosen all the bonds of human sympathy which hold society together, and to generate a wide-spreading demoralization, undermining the very foundations of the social fabric.

We have the authority of the author of this Report for believing it is perfectly possible, by legislative means, to remove and obliterate all these crying evils which it is even revolting to contemplate. Can there be a moment's doubt or hesitation on the part of the Government or the Legislature as to the imperious necessity of putting hand to the work, and blotting out, now and for ever, this foul stain on civilization and reproach to a Christian nation? We trust not—and, notwithstanding the Secretary of State for the Home Department has stated in his place in the House of Commons, that he is unwilling to deal with this subject, until he has before him the Report of the Commission of Health of Towns; yet we would fain hope—and earnestly trust, that unless this latter Report be certainly and speedily forthcoming, no speculative desire to make a comprehensive measure of amelioration will prevent the Government from applying themselves immediately to the removal of this vast and festering plague-sore, which has fixed itself as it were in the heart and entrails of the labouring population, utterly helpless and powerless to struggle against the evil which prostrates them, without the aid of a large and effective measure of interments, organized and watched by Government, with a jealous eye to the emancipation of the poorer classes from all the debasing and brutalizing horrors which this inquiry discloses.

We are assured all our readers will think, in the words of the Report, that—

"It were a reproach to the country, and its institutions and its government, and to its administrative capacity, to suppose that what is satisfactorily done in the German states may not, now that attention is directed to the subject, be generally done at least as well and satisfactorily in this country; or that the higher classes would not in whatever depends on their voluntary aid, exhibit as good and practical an example of community of feeling in taking a lead in the adoption of all arrangements tending to the common benefit, as that displayed in the states which have achieved the most satisfactory improvement of the practice of interment, by well-appointed officers of public health." 196.

We have not space to enter into any analysis of a large portion of the Report, which develops the aggregate and average expenses of funerals among the different classes of society, and the corruption and other specific effects on the living, incident to excessive charges for funerals—the organization of burial societies by undertakers, and their arbitrary proceedings—exemplifications of the economy of efficient preventive sanitary measures to diminish the expenses of funerals—examination of the obstacles to an early removal of the dead for interment—the nature of the establishments for the reception of the dead at Frankfort, Munich and other parts of Germany—evidence of the popular desire for improved and comprehensive arrangements in respect to interments in towns—and improvements that might be effected through such arrangements in respect to registration of the causes of death. On all these subjects there is much valuable matter, but of secondary importance to the great questions in relation to which they are brought forward.

The measures proposed by the Reporter for the removal of the evils to which we have adverted, may be briefly stated to consist in a plan for extra-mural burials to the exclusion of all interments in towns, and the appointment, under Government, of a staff of public functionaries to be termed officers of health, whose duties he defines to consist in verifying the fact and cause of deaths by personal attendance at the abodes of the deceased—calling for inquiry if he saw cause of suspicion or doubt—in giving assistance and information to the survivors—being prepared with a tariff of the prices of burial, and with instructions as to the regulations adopted for the public convenience, and for the more respectful performance of the ceremony of interment;—thus guarding the unprotected widow and the orphan from the harpies of undertakers who hang round the habitations of the dead as a vulture hangs round its prey—to victimize, by extravagant charges and extortion, the survivors. Thus it would appear it is proposed to empower these officers to take such a course as this. Speaking to the widow and survivor of the lowest class, he might say—

"The inspectors of public health have been empowered to regulate the practice and the charges for interment, and to contract for and on behalf of the public to ensure the means of burial in a proper and respectful manner for the highest, as well as for the most humble classes. Formerly, the charge for the funeral of a person of the condition in life of your husband was four or five pounds, but by the new regulations, an equally respectable interment is secured to you for little more than half the amount. You are, nevertheless, at liberty to obtain the means of burial from any private undertaker. You may also, if you prefer it, have burial in any private cemetery, or elsewhere." 162.

In the case of illness amongst the survivors, or of a death from epidemic disease, indicating an infected atmosphere, he might add—

"For the protection of your own health, and the health of your children and of your neighbours, it is requisite that the body be immediately removed to a place where it will be kept under the care of a physician, and inspected until the appointed time of interment, when it will be received by the friends and relations who attend." 163.

This assistance and these duties to be performed for the benefit of the survivors, and free of all expense, the officers being salaried by Government.

Without going more into detail respecting the proposed plans, we may say, that reserving some inconsiderable modifications, we think the whole scheme admirable, and worthy of the most serious attention. We heartily and earnestly desire to see these suggestions embodied in a practical measure, under the sanction of the Legislature.

We have a few words, however, to say on a subject in which we differ considerably from Mr. Chadwick—both in his estimate of the labour, and expense of health and time, which the duties he has sketched out for the officers of health will entail; and the salaries which he conceives will form an adequate remuneration for such services. In this matter, and we say it with regret and reluctance, the Barrister at Law has alone presided, and his estimate seems to us to have rather been dictated by the solution of the question of the smallest possible expense at which educated medical men might be induced to sacrifice their career as practitioners and all hopes of fortune or advancement, to devote their talents, and risk health and life in carrying out the details of the most important measure of public health and polity which this century has produced—rather than a consideration of what would be a fair and adequate remuneration to such men, conscientiously discharging duties at once onerous, painful and hazardous, and of the utmost importance to the safety and welfare of society.

If Mr. Chadwick, whose attention has been directed to the scale of remuneration for medical men in the army and navy, had not been limited by the view we have adverted to, he could scarcely, with the example before him, that the Government consider the medical officers, who have the direction of the department of the health of the army and of the navy respectively, worthy of emoluments approaching, if we are not mistaken, £2000. per annum, have sought to fix the remuneration of the officer who was to undertake the direction of the department of the health of the Metropolis, and more especially of a labouring population of near a million souls, at £657. with the privilege of driving a single-horse vehicle in the discharge of his lugubrious duties, at the public expense, on the principle that this would be good economy ! Still less would he, proceeding downwards from this liberal maximum, have classed 10 officers, on whom the whole labour of working out the detail of the systems would follow, at the respective rates of staff and regimental surgeons.

These officers, be it remembered, are not to enter into private practice, they are to be subject to calls throughout their districts, to proceed at any hour of the day, if not night, to verify the cause of death—to go into the chamber of death—into scenes of wretchedness and infection—there, in in the midst of ignorance, prejudice, and squalid misery, to convey information, give advice, and possibly to exercise a summary power to cause the dead body to be removed from the unwilling relations, and direct various hygienic measures to prevent the spread of infection. Fifteen such visits the Barrister at Law calculates the officer may make each day of his life (as long as it may last under such discipline), and for the pay of a staff-surgeon—just £350. per annum, or, upon an average, 1*s.* 2½*d.* per visit.

Let us reflect for a moment what are the qualifications such an officer of health must possess, ably to discharge his duties, and which therefore the Barrister expects to obtain and adequately to remunerate by the specified salary.

It is contemplated that he should not only have competent medical knowledge obtained from an expensive education, and improved by experience in practice, to enable him to form a correct opinion on the various causes influencing health and disease, and occasioning death, and of the best mode, under adverse circumstances, of removing or lessening all deleterious influences that the means at his disposal will admit—but he must be a man of good judgment and knowledge of the affairs of the world, and of character. He must often be called upon to determine whether the reports of the circumstances and causes of death are true and worthy of credence, or whether just grounds may exist for distrust and inquiry under a coroner's inquest? If he fails in this, he fails in one of the most important functions of his office, and may, and undoubtedly would, entail many and serious evils where he was sent to convey comfort and assistance. Nor is this all—other qualifications are still required—he is to be sent to communicate with the most ignorant and prejudiced—if he goes with powers to coerce and no power within himself to convince and persuade, sharp and thorny will be his path to those habitations of the wretched and the poor, and slender benefit will attend his ministrations and assistance. Is it not evident that he must be possessed of no common or mediocre share of tact, prudence, and worldly knowledge to deal successfully and yet kindly with this class of the population. With the educated, official business may generally be smoothly transacted by a very ordinary man, and with exceedingly small expenditure of tact—it is not so with the lower classes, who, in proportion to their want of knowledge, require judgment and ability in those who would influence them for any good purpose—still more when the object contemplated runs counter to their strongest prejudices.

How different are these duties to the ordinary routine of officers of the army or navy! We will not say how different are the qualifications, for many there are in both those services, who possess them in an eminent degree; but few such, we firmly believe, will be found inclined to give up, in mature life, their comparatively easy and pleasant positions with the duties belonging to them, with all the chances of war and flood, for the emoluments of an Inspector of Public Health and the honors of a one-horse vehicle; even were it not more than probable that frequent sickness and a premature death would follow their exertions in the zealous discharge of duties, such as they are defined by the writer who estimates their salaries.

We conclude with a hope that Her Majesty's Government will speedily take the subject into consideration. The evil is great—the necessity for action urgent. We also trust that, when any measure is framing, the Government will be better advised than we conceive they are in the present instance, and allow no mistaken views of economy to interfere with the efficient working of their measure by the medical officers to whom it may be confided. Because, in our overstocked profession, members of it are often driven, from penury and failure, to grasp at appointments like drowning men at straws, and to undertake duties however inadequately remunerated, it is not for a nation or a government to take advantage of such circumstances, and endeavour to obtain services of so important a character, without a fair and adequate remuneration. In reference to those duties sketched out in the Report, whether civilians or medical officers of the navy or army be selected, we emphatically pronounce the scale of remuneration suggested by Mr. Chadwick to be inadequate, and therefore unjust, and calculated to put in jeopardy the success of any large and comprehensive measure of the nature proposed.

